# Marriage with Drunkards

**Published:** 1891-04

**Authors:** 


### MARRIAGE WITH DRUNKARDS.

The efforts to raise the poor and degenerate inebriate and his family are practically of no value as long as marriage with inebriates is permitted. Recently the legislature of the State of Victoria, in Australia, has passed a law which gives the wife the right of divorce if the husband is found to be an habitual drunkard. If after marriage she discovers that he is an inebriate she can also get a divorce. The husband can do the same with the wife if she is proved to be an inebriate. This is a clear anticipation of the higher sentiment which demands relief from the barbarous laws which would hold marriage with an inebriate as fixed and permanent.—*Journal of Inebriety.*

